# The asymmetric relationship of parental psychological flexibility and parenting involvement in autism: anxiety as a mediator

**DOI:** 10.3389/fpubh.2026.1912741

**Published:** 2026-09-16

**Authors:** Jingyi Liu, Zhiqin Chen, Jiayi Li, Dianzhi Liu, Xiajing Wei

**Affiliations:** 1 School of Urban Governance and Public Affairs, Suzhou City University, Suzhou, China; 2 Department of Rehabilitation, Suzhou BenQ Medical Center, Suzhou, Jiangsu Province, China; 3 School of Education, Soochow University, Suzhou, China

**Keywords:** anxiety, autism spectrum disorder, dyadic analysis, parental involvement, parental psychological flexibility

## Abstract

**Introduction:**

Parents of children with autism spectrum disorder (ASD) face immense pressure and challenges. In traditional Chinese culture, a distinct division of labor exists, with mothers typically bearing greater parenting burdens. This study examined the relationship between parental psychological flexibility (PPF) and parental involvement in families of children with ASD, investigated the mediating role of anxiety, and explored the interaction effects between parents.

**Methods:**

The study included 83 pairs of parents of children with ASD. Parental psychological flexibility, anxiety, and parenting involvement were assessed using the Parental Psychological Flexibility Scale, Anxiety Scale, and Parenting Involvement Scale, respectively. An actor-partner interdependence model was constructed.

**Results:**

Both paternal and maternal PPF exhibited significant actor effects on parental involvement, while maternal PPF showed a partner effect on paternal involvement. When anxiety was included as a mediator, maternal PPF was negatively associated with both maternal and paternal anxiety. There was also a significant indirect association between maternal PPF and paternal involvement through maternal anxiety.

**Discussion:**

These findings suggest that PPF is a core factor contributing to parenting. In the Chinese cultural context, enhancing mothers' psychological flexibility may be key to improving family parenting capacity.

## Introduction

Autism spectrum disorder (ASD) is a neurodevelopmental disorder characterized by difficulties in social communication and stereotypical behavior (APA, 2013). According to the latest 2025 report from the US Centers for Disease Control and Prevention, the prevalence of ASD has reached one in 31 cases, with a continuing upward trend. Following an ASD diagnosis, parents typically assume long-term functional caregiving responsibilities that negatively affect their physical and mental health (1, 2). As a positive psychological resource, parental psychological flexibility (PPF) serves as an effective protective factor against long-term psychological burden (3, 4). PPF helps parents effectively address parenting challenges and fosters healthy parent-child relationships. With the development and validation of Acceptance and Commitment Therapy (ACT), PPF has been demonstrated to positively affect families with children with ASD by enhancing parental mental health and family functioning (5).

Therefore, research on families with children with ASD should be conducted in a broader cultural context. In China's cultural and social framework, traditional beliefs hold that children with disabilities cause stigma to the family (6), which imposes additional psychological burden on parents of children with ASD. More than 90% of families with children with ASD in China experience impaired quality of life and dysfunction (China Association of Persons with Mental Disabilities and Their Relatives and Friends, 2014). Furthermore, shaped by collectivist cultures, Chinese families exhibit distinct role differentiation between parents in caregiving responsibilities and emotional coping (7). This manifests as fathers shouldering greater economic responsibilities while mothers taking on more childcare duties. Given the long-term care needs of children with ASD, this pattern may be amplified in families. Notably, studies on ASD have long overemphasized maternal roles, as exemplified by the refrigerator mother hypothesis, which attributes ASD causation to maternal emotional coldness. Although this hypothesis has been thoroughly disproven by advances in cognitive neuroscience, its enduring influence has led to mothers being disproportionately emphasized in ASD research and interventions, whereas fathers' roles remain relatively unexplored (8). Recent research perspectives have gradually shifted from the individual to the family system, suggesting that parental psychological resources (e.g., PPF) may jointly shape families with children with ASD in distinct ways. In this context, clarifying how the protective factor of PPF contributes to parenting practices among parents of children with ASD and how this relationship is reciprocally shaped between parents of children with ASD has significant theoretical and practical implications.

### PPF and parental involvement

PPF emphasizes parents' ability to maintain an accepting attitude toward negative emotions encountered while practicing values-aligned parenting behaviors (9). As a vital psychological resource, PPF profoundly shapes the level of parental involvement in the daily caregiving of children with ASD (10). Exploring parental involvement is particularly crucial in families with children with ASD because these children require long-term intensive care from their parents (11). Parental involvement refers to the cognitive, emotional, and behavioral involvement of either the father or mother in the process of raising a child to promote healthy development (12). Recent studies indicate that approximately 54.3% of mothers of children with ASD resign from their jobs to care for their children, whereas only 2.8% of fathers do so (Wang et al., 2024). Additionally, fathers spend less time on childrearing and experience lower levels of stress than mothers. Fathers also play an irreplaceable role (6), although parental involvement dimensions may differ from those of maternal involvement (12). As children with ASD experience frequent difficulties with emotional regulation, social avoidance, and behavioral challenges, daily care demands substantial time and emotional energy to sustain consistent interactions and interventions (8). Parents who maintained an open, accepting, and flexible psychological state when faced with these pressures were more likely to engage in positive parenting. Conversely, parents with low PPF were more prone to experiential avoidance (Fonseca et al., 2020), which diminished the breadth and depth of parenting involvement, and might even negatively affect the child.

### Mediating role of anxiety

Anxiety is one of the most common emotional disturbances among parents of children with ASD, with parents of children with ASD consistently reporting significantly higher anxiety levels than those of typically developing children (13). ACT theory proposes that parental PPF serves as an individual emotional regulation resource to cope with negative experiences associated with parenting (9). Parents with high PPF can respond to their children's unstable negative emotions and challenging behaviors with openness and acceptance rather than attempting to alleviate discomfort through suppression or avoidance (14). Studies have shown that PPF in children with ASD is negatively correlated with negative emotions, such as anxiety (15). Parental anxiety is also associated with parental involvement, which reduces positive responsiveness during interactions with children. Related research has indicated that parents' capacity to provide optimal nurturing is influenced by their emotional state, with higher parental distress associated with fewer supportive behaviors (16). Parenting children with ASD often requires high levels of patience and emotional regulation. Under prolonged high-stress conditions leading to anxiety, parents of children with ASD tend to reduce their involvement (6). However, previous findings have not been entirely consistent, with one study reporting no significant correlation between mothers' anxiety and parental involvement (17). This suggests that the relationship may vary according to parental gender roles. Thus, anxiety may mediate the association between parental stress and parenting behaviors in families with children with ASD, with potential differences between fathers and mothers.

### Differences between fathers and mothers of children with ASD

Although studies have increasingly recognized fathers' roles in families, these roles remain largely overlooked in families of children with ASD. This was partly because mothers often serve as primary caregivers in families with children with ASD, and there are challenges in collecting data from fathers of children with ASD (18). The crossover hypothesis in family systems theory proposes that the family is a dynamic, interconnected system in which the experiences, emotions, and behaviors of one family member can influence all others in the household (19). Evidence suggests that crossover effects in families become more pronounced under chronic stress conditions (20), making the focus on co-parenting relationships in families of children with ASD particularly crucial.

Influenced by the traditional Chinese family culture, parents of children with ASD exhibit differences in their mental health and parenting practices (21). First, mothers of children with ASD often invest more time and energy in daily care and rehabilitation training, whereas fathers tend to participate to a relatively limited extent (21). In single-parent or separated families, children predominantly live with their mothers, further highlighting the central role of mothers in caregiving (22). Second, cultural background may lead to different emotional and psychological coping strategies among parents of children with ASD. Following diagnosis, traditional cultural expectations for mothers to “devote themselves entirely to childcare” make them more likely to internalize caregiving responsibilities as personal obligations. This results in heightened emotional stress and psychological distress, causing mothers to feel the impact of their child's condition more intensely than fathers. Mothers perceive the effect of raising children with autism on their quality of life as significantly greater than fathers do (2) and report shouldering more caregiving responsibilities (23). They often exhibit emotional issues, such as anxiety and depression, emotional distress, and other negative outcomes (24, 25). By contrast, fathers in the Chinese culture primarily shoulder economic responsibilities, engage less directly in caregiving, and are less likely to proactively seek family support or professional resources (26). This disparity may stem from the culturally assigned, more intensive, and sustained caregiving roles for mothers. Therefore, understanding how parental interactions influence each other during the parenting process is crucial. Specifically, how PPF among fathers and mothers of children with ASD interacts with parental involvement remains unclear, and whether or how this relationship is mediated by anxiety remains to be determined.

Accordingly, this study aimed to explore the following in parents of children with ASD: (1) the relationship between PPF and parental involvement and whether there was a mutual relationship between fathers and mothers; and (2) the mediating role of anxiety in the relationship between paternal and maternal PPF and parental involvement and the bidirectional relationship between fathers and mothers.

## Methods

### Participants

This study recruited participants through a psychological association and an autism research center at a university in China, employing a combination of convenience sampling and snowball sampling. The recruitment channels include: disseminating research recruitment information via parent support groups and the WeChat Official Account; inviting families who have already participated in the study to recommend this research to other eligible parents of children with ASD. Enabling interested parents to join the group by scanning a QR code, after which the researchers will conduct individual eligibility screenings. These families originate from Multiple regions in China, encompassing various economic and cultural conditions. All the couples were parents (mothers and fathers from the same family) nof children aged 4–12 years, with a clinical record of DSM-IV-TR diagnosis of ASD from qualified psychiatrists. Excluded families in which the child had concurrent organic diseases (4 families), as well as only one parent completed the questionnaire (17 families). finally, 83 pairs (166 participants) of parents of children with autism participated in this study.

All parents completed the Parental Psychological Flexibility Questionnaire and Anxiety Questionnaire. And both parents completed the Paternal Involvement questionnaire on different versions of the questionnaire: Fathers completed the Paterntal Involvement Questionnaire, while mothers completed the Mother Parental Involvement Questionnaire. Among the participants, 68.7% of mothers were full-time caregivers, and 2.4% of fathers were full-time caregivers. The study was approved by the University ethics committee.

### Measures

#### Parental psychological flexibility

Parental Psychological Flexibility was measured by the Parental Psychological Flexibility Questionnaire (PPFQ), which developed by Burke and Moore (9). The revised version has been widely used in Chinese families and demonstrated good reliability and validity (15). The PPFQ including 16 items comprises three dimensions: cognitive defusion (e.g., “My emotions prevent me from being the perfect father/mother I envision”), acceptance (e.g., “No matter how I feel, it does not affect how I treat my child”), and committed action (e.g., “I do not allow my child to do things that worry me”). Responses are rated on a 7-point Likert scale (1 = Never, 7 = Always), with higher scores indicating greater psychological flexibility in parenting. The Cronbach's α coefficients in this study was 0.857 for fathers and 0.910 for mothers.

#### Anxiety

Anxiety was measured by the Self-rating Anxiety Scale (SAS), developed by Zung (27). The scale consists of 20 items rated on a 4-point scale (1 = never, 4 = all the time). Higher scores indicate more severe anxiety. In this study, the Cronbach's α coefficients for paternal/maternal anxiety were 0.745 and 0.808, respectively.

#### Paternal involvement

Paternal involvement was assessed by using Paternal Involvement Questionnaire. Wu et al. (12) revised the questionnaire development by Lamb's to align with Chinese cultural characteristics, comprehensively reflecting Chinese paternal involvement. The questionnaire had widely used in Chinese parents and has good reliable and vaild in structure, which was suitable in Chinese families (28). In this study, fathers self-reported their involvement using this 56-item questionnaire, which measures three dimensions: interactivity (e.g., “I make time to talk with my child”), accessibility (e.g., “I pick up and drop off my child”), and responsibility (e.g., “I set an example for my child through my own actions”). The scale employs a 5-point rating (0 = never, 4 = always), with higher scores indicating greater paternal involvement in childrearing.

Considering the cognitive realities of children with ASD, we invited five experts to independently rate the applicability of the scale items for ASD families. Items with I-CVI below 0.78 were deleted: Item 33 (“I monitor my comments about social phenomena in front of my child”), Item 36 (“I help my child set life goals and future directions”), Item 45 (“I discuss my child's learning difficulties with them”), and Item 46 (“I teach my child to take responsibility for their own affairs”). The revised scale demonstrated good content validity, with an S-CVI/Ave of 0.92 and all remaining items achieving I-CVI values of 0.80 or above. The Cronbach's α coefficient for this study was 0.928.

#### Maternal involvement

*Maternal involvement* assessed by using Mother Involvement in Parenting Questionnaire, developed by Guo and revised by Hou (29). This 23-item scale measures maternal involvement across four dimensions: daily care (e.g., take care of the child's rest and sleep.), learning stimulation (e.g., eading picture books to the child), behavioral discipline (e.g., helping the child establish daily routines), and warm encouragement (e.g., praising the child's good behavior). Responses were rated on a 4-point scale (1 = never, 4 = always), with higher scores indicating greater maternal involvement in childrearing. The Cronbach's α coefficient for this scale in this study was 0.873.

### Data analysis

Data processing was conducted using SPSS 25.0, including descriptive statistics, correlation analysis, and common method bias testing. Mplus 8.0 was used to construct actor-partner interdependence models (APIMs) for PPF and parenting involvement. Subsequently, parental anxiety was incorporated to build actor-partner interdependence mediation models (APIMeMs), with mediation effects tested via 5,000 bootstrap samples. For the mediation analysis, statistical significance was determined based on the 95% bootstrap confidence interval, with an indirect effect considered significant when the interval did not include zero. The APIMs allowed us to simultaneously test how one parent's predictors influence their own (actor effects) and their partners (partner effects), while also testing mediation effects within this dyadic relationship. This methodology has been widely applied in family research, including family studies among autism (6, 21).

## Results

### Common method bias

Common method bias was examined using Herman single-factor test. Results indicated that the first factor explained 27% of total variance, below the 40%, which suggested no common method bias in this study.

### Preliminary analysis

Descriptive statistics and Pearson correlations for all variables were presented in Table 1. Correlation analysis revealed that maternal anxiety was negatively correlated with maternal involvement, paternal involvement, and maternal PPF. Paternal involvement was positively correlated with maternal PPF. Maternal involvement was positively correlated with maternal full-time employment, and paternal involvement was positively correlated with paternal full-time employment.

**Table 1:** Descriptive statistics and Correlations of the primary variables in the study.

Variable	*M*	*SD*	1	2	3	4	5	6	7
1 Maternal involvement	3.06	0.62	–						
2 Paternal involvement	3.74	0.29	0.20	–					
3 Maternal PPF	4.50	1.08	0.24	0.31^**^	–				
4 Paternal PPF	4.76	0.85	0.13	0.21	0.15	–			
5 Maternal anxiety	1.86	0.43	−0.29^**^	−0.42^**^	−0.54^**^	−0.01	–		
6 Paternal anxiety	1.73	0.36	−0.15	−0.36	−0.33^**^	−0.26^*^	0.50^**^	–	
7 Maternal full–time caregiver	–	–	0.34^**^	−0.06	0.11	−0.01	0.17	0.16	–
8 Paternal full–time caregiver	–	–	0.07	0.28^*^	0.17	0.16	0.04	−0.07	−0.07

Maternal PPF = Maternal parental psychological flexibility, Paternal PPF= Paternal parental psychological flexibility. Maternal/paternal full-time caregiver represented mother/fathers stays home full-time to care for the children. Full-time childcare were coded as 1, part-time childcare were coded as 0. ^*^p < 0.05, ^**^p < 0.01.

### APIM analyses

An APIM was constructed with maternal/paternal parenting psychological flexibility as the independent variable, maternal/paternal involvement as the dependent variable, and controlling for whether the mother/father was a full-time caregiver. The model fit well: χ^2^*/df* = 1.15, RMSEA = 0.043, CFI = 0.975, TLI = 0.962, SRMR = 0.065. Results indicated: (1) Both parents showed actor effects. Maternal PPF significantly contributed maternal involvement (β = 0.26, *p* = 0.015); paternal PPF significantly contributed paternal involvement (β = 0.21, *p* = 0.029). (2) A direct partner effect was observed for mothers: maternal PPF significantly contributed paternal involvement (β = 0.33, *p* < 0.001). As shown in Figure 1.

**Figure 1:**
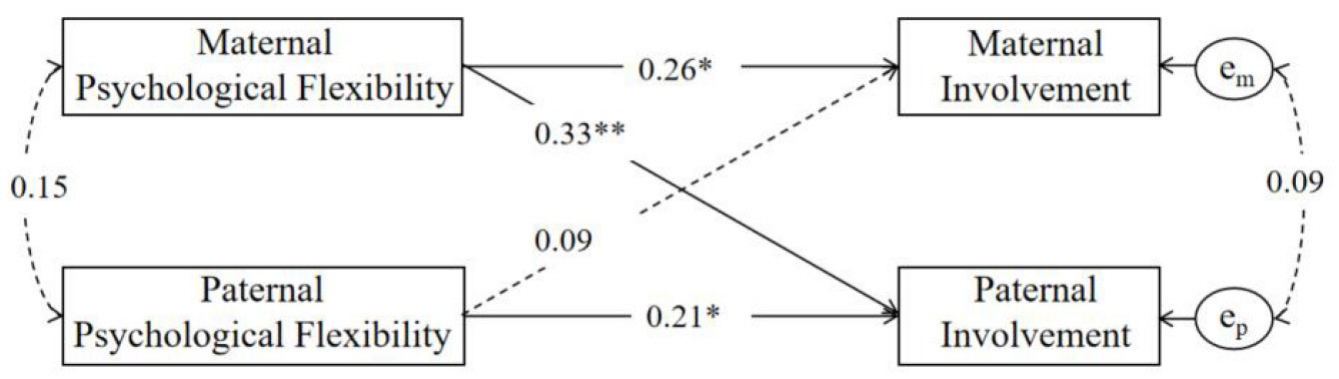
Actor–partner interdependence model between maternal/paternal PPF and maternal/paternal involvement. Note: Actor paths represent within-parent associations, whereas partner paths represent cross-parent associations. Values shown are standardized path coefficients. Arrows indicate the specified model paths. Control variables (maternal/paternal full-time caregiver) were not shown in the figure to simplify the model, *N* = 83 pairs, *p < .05, **p < .01,

### APIMeM analyses

An APIMeM was constructed based on the direct relationship between maternal/paternal PPF and maternal/paternal involvement, with maternal/paternal anxiety as the mediating variable, while controlling for whether the parent was a full-time caregiver. The model fit was good: χ^2^*/df* = 1.33, RMSEA = 0.063, CFI = 0.972, TLI = 0.931, SRMR = 0.049. Results indicated that after incorporating mediating variables, maternal PPF significantly and negatively related both maternal anxiety (β = −0.55, *p* < 0.001) and paternal anxiety (β = −0.29, *p* < 0.001). Maternal anxiety further significantly and negatively related paternal involvement (β = −0.24, *p* = 0.048). An actor effect of father was observed: paternal PPF negatively related the father's own anxiety (β = −0.22, *p* = 0.041) and positively contributed the father's own involvement (β = 0.20, *p* = 0.051). A 5,000-sample bootstrap analysis examined the mediating role of maternal PPF → maternal anxiety → paternal involvement. The indirect effect was 0.134 [*p* =.053, 95% bootstrap CI (0.014, 0.287)] and was considered statistically significant according to the bootstrap confidence-interval criterion because the interval did not include zero.

## Discussion

This study used paired father-mother data from children with ASD to construct an actor-partner interdependence mediation model. It examined the role of anxiety in mediating the relationship between PPF and parental involvement in parents of children with ASD, while also exploring sex differences in these pathways. The findings contribute to our understanding of the asymmetric role of PPF between fathers and mothers of children with ASD in the traditional Chinese culture and deepen our understanding of how parental anxiety is associated with parental involvement in such families, providing a foundation for more precise future interventions.

### PPF plays a central role in parenting

This study found that PPF was significantly and positively associated with parental involvement (see Figure 1) and significantly and negatively associated with their anxiety levels (see Figure 2). This result supports ACT theory, which proposes that, as a positive psychological resource, PPF serves as an effective protective factor against long-term psychological burdens (4) and is a key psychological resource for positive parenting behaviors (9). Parenting children with ASD often involves unpredictable behavioral and emotional challenges and demands sustained self-regulation and emotional management from parents. Parents with higher PPF perceived these difficulties as challenges rather than threats. They engage in daily rehabilitation and companionship with their children with ASD with greater acceptance, whereas commitment to action enhances their capacity for effective intervention, thereby sustaining high levels of parental involvement. Regarding anxiety, our findings align with previous research: PPF negatively correlates with negative emotions such as anxiety (15). This may stem from the cognitive dissociation and acceptance dimensions of PPF, which enable parents to separate themselves from their emotions and cease to resist the challenges posed by their children with ASD (14), thereby facilitating effective anxiety regulation (9). These findings suggest that enhancing PPF is an effective approach to improve anxiety levels among parents of children with ASD (13).

**Figure 2:**
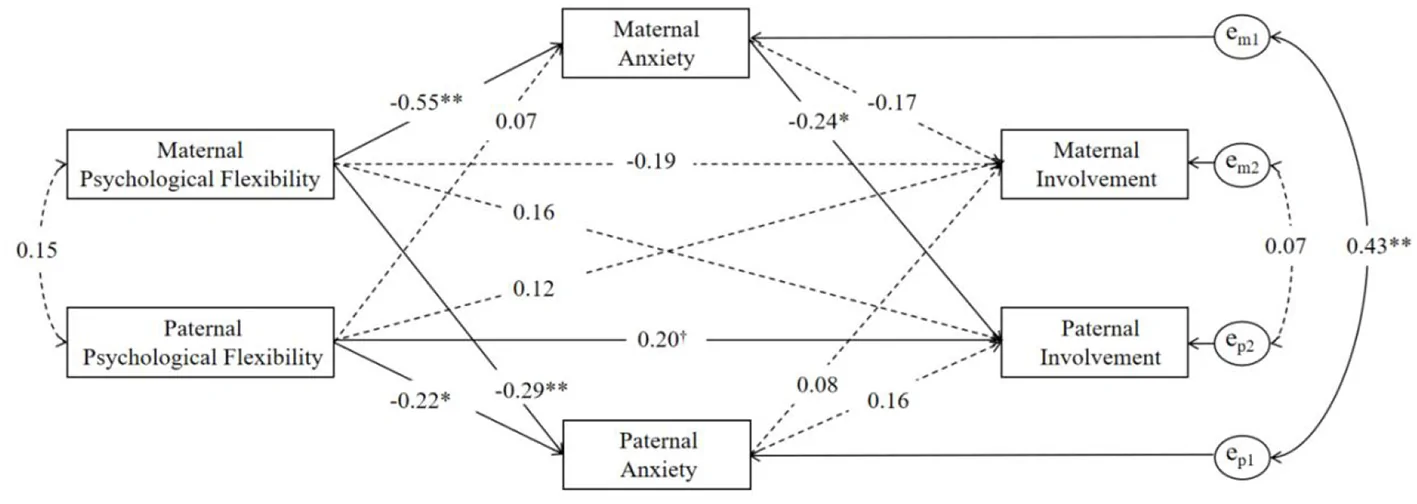
Actor–partner interdependence mediation model of maternal/paternal PPF and maternal/paternal involvement mediated by parental anxiety. Note: Actor paths represent within-parent associations, whereas partner paths represent cross-parent associations. Values shown are standardized path coefficients. Arrows indicate the specified model paths. Control variables (maternal/paternal full-time caregiver) were not shown in the figure to simplify the model, *N* = 83 pairs, †*p* < 0.06, *p < .05, **p < 0.01.

### Mothers play a dominant parenting role

This study found that mothers contributed to fathers through multiple unidirectional pathways: First, maternal PPF was positively associated with paternal involvement (see Figure 1) and negatively related paternal anxiety (see Figure 2). Second, maternal anxiety significantly negatively related paternal involvement (Figure 2), whereas no reciprocal effect was observed in fathers. This finding suggests suggests asymmetric cross-parental interactions among Chinese families. This unidirectional pattern underscores the dominant role of mothers in the parenting process of families. There are two potential reasons for this finding.

First, maternal PPF has crucial family protective functions. Mothers with high parenting flexibility exhibit open, accepting, and adaptable attitudes toward their children's behavioral challenges and better tolerate daily parenting difficulties (9). Due to families of children with ASD face more challenging parenting circumstances, maternal PPF not only benefits children directly but also reduces household anxiety, provide positive modeling and emotional security for fathers, thereby enhancing their willingness and stimulate positive parenting engagement, which highlights the mother's dominant parenting role. These findings suggest that interventions for families of children with ASD should prioritize the cultivation and support of maternal PPF. Improving maternal parenting styles not only enhances parenting quality but also yields secondary benefits for fathers and overall family functioning.

Second, the findings showed the mediating role of maternal anxiety in families of children with ASD, indicating that maternal anxiety associated with paternal involvement in child-rearing. This may be due to maternal anxiety not only concerns the individual's internal experience but also becomes a transmissible pathway for emotional contagion between partners in Chinese families (30). Given the transmission of emotions between spouses (19). In families of children with ASD, mothers' emotional stability is particularly important, as it fosters a more secure emotional environment, and lower emotional burden among fathers. This may alleviate fathers' shame and guilt related to raising a child with ASD, thereby breaking their self-protective cycle of anxiety avoidance and reduced parental involvement (31), making fathers more willing or capable of participating in caregiving. The discovery of this emotional chain highlights maternal emotional health as a critical node in families of children with ASD. Also traditional Chinese cultural norms emphasizing male external responsibility and female internal management, and consequently, mothers of children with ASD shoulder an overwhelming majority of their caregiving duties. Mothers of children with ASD are more likely to resign from their jobs to care for their children (32), Consistent with this finding, 68.7% of the mothers in our sample were full-time caregivers, which also indicates that mothers play a dominant parenting role. It is worth noting that maternal and paternal involvement were measured with different scales, reflecting distinct parenting role expectations: Fathers are expected to contribute in multidimensional but non-mandatory ways such as interactivity, accessibility, and responsibility, whereas mothers are expected to perform irreplaceable daily care tasks such as daily care, learning stimulation, behavioral discipline, and warm encouragement. This distinction reinforces the irreplaceable foundational role of mothers in Chinese ASD families, which underpins the stability of the entire family parenting system. Besides, mothers bear significantly more anxiety than their fathers, imposing invisible negative effects on the entire family. Under chronic stress conditions in families of children with ASD, focusing on coparenting relationships is particularly crucial.

The TD studies reviewed here did not find a clear mother-dominant pattern. Beck et al. (33) found that parents' psychological flexibility was related to lower parenting stress and better later coparenting. These associations were found only within parents, and no partner effects were found. Li et al. (7) followed 700 Beijing families for 2 years. Parenting stress predicted both parents' own and their partners' parent-child relationships. Mindful parenting mediated these actor and partner effects. The effects did not differ between mothers and fathers. However, the present study found a different pattern. Maternal PPF was associated with paternal anxiety and involvement. Maternal anxiety was also associated with paternal involvement. No corresponding pathways from fathers to mothers were found. These results suggest that mothers may play a dominant role in the parenting process of Chinese families of children with ASD. This asymmetric mother-to-father pattern is an important contribution of the present study.

### Maternal involvement exhibits stability

Results showed that maternal involvement commitment remained unaffected by the mother's own anxiety or the father's involvement. First, this may stem from traditional Chinese cultural influences, in which the “mother” role is strongly constructed as a selfless caregiver. Compared with fathers, mothers bear a heavier moral burden and internalize the long-term responsibility of caring for and addressing their children's challenges. This leads mothers to believe that they must fulfill their parenting responsibilities regardless of their personal state, leading them to maintain or even intensify their commitment despite emotional distress. Second, In Chinese families, the traditional gender division of labor (men handle external affairs, women manage domestic matters) positions women as primary caregivers. Accordingly, most mothers in families of children with ASD quit their jobs and perceive caregiving as a job (32). Thus, irrespective of their emotional condition or paternal participation, mothers must complete their caregiving tasks. Third, a study has pointed out that mothers in Chinese families of children with ASD demonstrate remarkable resilience that enables them to sustain their caregiving involvement, by accepting their child's condition, adhering to responsibility-driven care, and finding hope and meaning in their parenting journey (34). The stability of maternal involvement stems not only from a sense of duty and gendered division of labor, but also from the narrative of maternal and feminine acceptance of adversity and self-growth in the face of challenges. Finally, Maternal involvement remains unrelated to fathers, partly because mothers hold a dominant position in family upbringing and partly because women's emotions are more outwardly expressed and fluctuate more readily, directly shaping the family atmosphere and interaction patterns. Fathers' parenting motivation tends to be more “action-oriented,” meaning they participate primarily through concrete actions rather than emotional expression, often suppressing their own emotions (35). Consequently, fathers' emotions are typically not transferred to mothers, creating a reverse effect.

In summary, in Chinese families, mothers of children with ASD play a pivotal role in child-rearing. Their PPF and anxiety levels play a critical role in shaping overall family parenting, with their effects on fathers serving. Cultivating mothers' psychological flexibility and alleviating their anxiety are keys to enhancing the quality of family parenting.

This study has several limitations. First, the modest sample of 83 parent dyads was recruited through convenience sampling in China. This may have introduced selection bias and limited sample representativeness. Moreover, the cultural explanations were not directly tested. The findings should therefore be generalized beyond Chinese families with caution. Second, only full-time caregiving status was controlled. Potential confounders, such as child age, ASD severity, socioeconomic status, parental education, and time since diagnosis, should be considered. Third, all variables were measured by parental self-report. Social desirability, recall bias, and common method bias cannot be fully excluded, despite the Harman single-factor test. Finally, the cross-sectional design prevents causal conclusions. Future studies should use larger and more diverse samples, multiple assessment methods, and longitudinal or cross-cultural designs.

## Conclusion

Under the conditions of this study, the following conclusions are drawn:

PPF is a key factor that promotes parental involvement in children with ASD.

In China, maternal PPF can reducing maternal anxiety, promoting paternal involvement in child-rearing, and alleviating paternal anxiety. Maternal psychological flexibility and emotions play a critical role in overall family parenting, with their effects on fathers representing a central pathway.

Our findings highlight maternal psychological flexibility as a key target for ASD family interventions. These findings indicate that ASD family interventions should take the enhancement of maternal PPF as a core strategic focus. Rehabilitation services should include psychological support, respite care, and career development for primary caregivers (especially mothers), along with policy incentives to promote paternal involvement.

## Data Availability

The raw data supporting the conclusions of this article will be made available by the authors, without undue reservation.
